# Potential utility of l-carnitine for preventing liver tumors derived from metabolic dysfunction–associated steatohepatitis

**DOI:** 10.1097/HC9.0000000000000425

**Published:** 2024-04-12

**Authors:** Junyan Lyu, Hikari Okada, Hajime Sunagozaka, Kazunori Kawaguchi, Tetsuro Shimakami, Kouki Nio, Kazuhisa Murai, Takayoshi Shirasaki, Mika Yoshida, Kuniaki Arai, Tatsuya Yamashita, Takuji Tanaka, Kenichi Harada, Toshinari Takamura, Shuichi Kaneko, Taro Yamashita, Masao Honda

**Affiliations:** 1 Department of Clinical Laboratory Medicine, Kanazawa University Graduate School of Medical Sciences, Kanazawa, Japan; 2 Department of Gastroenterology, Kanazawa University Graduate School of Medical Sciences, Kanazawa, Japan; 3 Research Center of Diagnostic Pathology, Gifu Municipal Hospital, Gifu, Japan; 4 Department of Human Pathology, Kanazawa University Graduate School of Medical Sciences, Kanazawa, Japan; 5 Department of Endocrinology and Metabolism, Kanazawa University Graduate School of Medical Sciences, Kanazawa, Japan

## Abstract

**Background:**

Recent reports have unveiled the potential utility of l-carnitine to alleviate metabolic dysfunction–associated steatohepatitis (MASH) by enhancing mitochondrial metabolic function. However, its efficacy at preventing the development of HCC has not been assessed fully.

**Methods:**

l-carnitine (2 g/d) was administered to 11 patients with MASH for 10 weeks, and blood liver function tests were performed. Five patients received a serial liver biopsy, and liver histology and hepatic gene expression were evaluated using this tissue. An atherogenic plus high-fat diet MASH mouse model received long-term l-carnitine administration, and liver histology and liver tumor development were evaluated.

**Results:**

Ten-week l-carnitine administration significantly improved serum alanine transaminase and aspartate transaminase levels along with a histological improvement in the NAFLD activity score, while steatosis and fibrosis were not improved. Gene expression profiling revealed a significant improvement in the inflammation and profibrotic gene signature as well as the recovery of lipid metabolism. Long-term l-carnitine administration to atherogenic plus high-fat diet MASH mice substantially improved liver histology (inflammation, steatosis, and fibrosis) and significantly reduced the incidence of liver tumors. l-carnitine directly reduced the expression of the MASH-associated and stress-induced transcriptional factor early growth response 1. Early growth response 1 activated the promoter activity of neural precursor cell expressed, developmentally downregulated protein 9 (NEDD9), an oncogenic protein. Thus, l-carnitine reduced the activation of the NEDD9, focal adhesion kinase 1, and AKT oncogenic signaling pathway.

**Conclusions:**

Short-term l-carnitine administration ameliorated MASH through its anti-inflammatory effects. Long-term l-carnitine administration potentially improved the steatosis and fibrosis of MASH and may eventually reduce the risk of HCC.

## Graphical abstract

**Figure:**
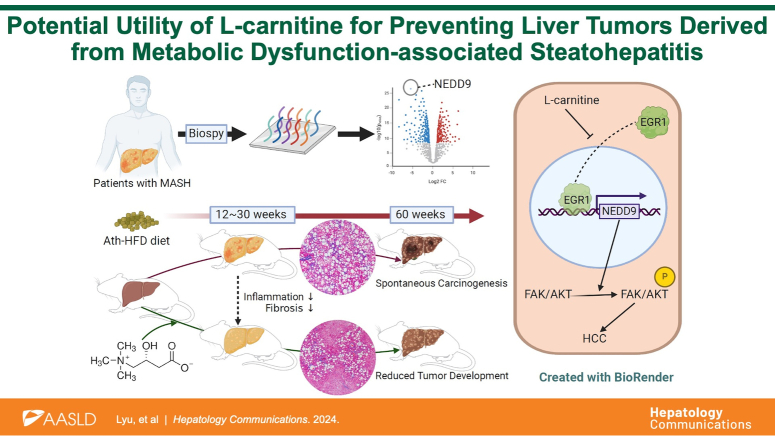

## INTRODUCTION

“Steatotic liver disease” is the newly proposed overarching term for metabolic dysfunction–associated steatotic liver disease (MASLD), replacing NAFLD, while metabolic dysfunction–associated steatohepatitis (MASH) is the replacement term for NASH.^1^

MASLD/MASH has emerged as one of the most prevalent liver conditions globally, posing a significant and escalating health burden. Especially, the incidence of MASH-related hepatocellular carcinoma (MASH-HCC) has been steadily increasing and it now constitutes a substantial portion of HCC cases. However, despite its growing prevalence, the pathogenesis of MASH-HCC remains enigmatic and effective therapeutic strategies are limited.

l-trimethyl-3-hydroxy-ammoniabutanoate, commonly known as l-carnitine, is an amino acid derivative that has a pivotal role in orchestrating a multitude of essential biological processes.^2^,^3^,^4^ l-carnitine plays a central role in mitochondrial function, facilitating fatty acid metabolism, and contributing to ATP production. l-carnitine deficiency can give rise to various metabolic disorders, including diabetes, cardiovascular diseases, kidney diseases, and polycystic ovarian syndrome.^5^ Recent reports have unveiled the potential utility of l-carnitine to alleviate MASLD by enhancing mitochondrial metabolic function, promoting the β-oxidation of free fatty acids, and reducing oxidative damage in liver cells.^6^,^7^,^8^,^9^ Clinical studies and meta-analyses have further suggested that l-carnitine administration can significantly improve serum alanine transaminase (ALT) and aspartate transaminase (AST) levels as well as NAFLD activity scores in patients with MASH.^10^

Although several mouse models and clinical data have shown that l-carnitine potentially improves the metabolic abnormalities and liver dysfunction induced by MASH, its efficacy in preventing the development of HCC has not been fully addressed. Previously, we established a MASH-derived HCC mouse model by feeding mice an atherogenic plus high-fat diet (Ath+HFD) for 68 weeks. Ath+HFD mice develop steatosis, inflammation accompanied by ballooned hepatocytes, fibrosis, and eventually HCC at a high frequency, which closely mimic the progression of human MASH-derived HCC.^11^,^12^

In the present study, we showed that l-carnitine significantly improved the pathophysiology of MASH in human and mouse livers. We also demonstrated that long-term l-carnitine administration substantially suppressed the development of liver tumors in Ath+HFD MASH model mice. Our data imply the potential utility of l-carnitine for treating MASH and preventing the subsequent occurrence of HCC, suggesting the value of performing large-scale clinical trials of l-carnitine for MASH.

## METHODS

### Patients

**FIGURE 1:**
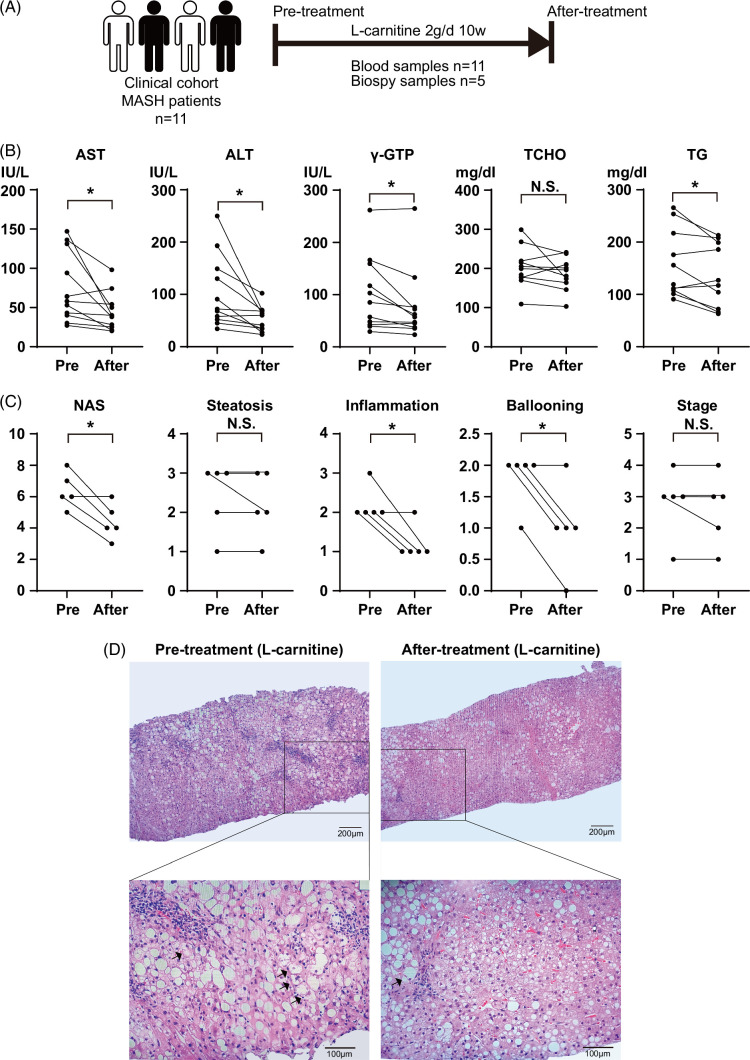
l-Carnitine improves liver inflammation in patients with MASH. (A) Eleven patients with MASH were enrolled in the clinical study. Blood serum (n=11) and liver biopsy (n=5) samples were collected before and after l-carnitine administration. (B) Changes in the serum levels of AST, ALT, γ-GTP, TCHO, and TGs induced by l-carnitine administration. (C) Changes in the histological features of liver biopsy samples induced by l-carnitine administration. (D) Representative hematoxylin and eosin staining of liver biopsy samples before and after l-carnitine administration. The black arrows indicate hepatocellular ballooning. Scale bar: 100 μm. Data are presented as the mean±SD. *p<0.05, NS, not significant. Abbreviations: ALT, alanine transaminase; AST, aspartate transaminase; γ-GTP, gamma-glutamyl transferase; MASH, metabolic dysfunction–associated steatohepatitis; NAS, nonalcoholic fatty liver disease activity score; TCHO, total cholesterol; TG, triglyceride.

Eleven patients who were histologically proven as MASH by liver biopsy were enrolled in this study (Figure 1A and Supplemental Table S1, http://links.lww.com/HC9/A845). We excluded all other liver disorders in each patient. All participants reported drinking <20 g/day ethanol. All participants were administered 2 g/day l-carnitine (two 500 mg l-carnitine tablets twice daily) for 10 weeks, and blood liver function tests and lipid profiles were monitored. Serial liver biopsies were taken from five of the patients for histological evaluations (Figure 1A and Supplemental Table S1, http://links.lww.com/HC9/A845). A single pathologist (Kenichi Harada), who was blinded to both the clinical information (eg, treatment assignments, participant characteristics, and the order in which the biopsy specimens were obtained), histologically evaluated all biopsy specimens. The biopsied tissues were scored for steatosis (from 0 to 3), stage (from 0 to 4), and grade (from 0 to 3), as described,^13^,^14^ according to the standard criteria of Brunt et al.^15^ The NAFLD activity score was calculated as the unweighted sum of the scores for steatosis (0–3), lobular inflammation (0–3), and hepatocellular ballooning (0–2).^16^ The research protocols were approved by the Human Genome/Gene Analysis Research Ethics Committee of Kanazawa University and its related hospitals and the study was conducted in accordance with the Declarations of Helsinki and Istanbul. Written informed consent was obtained from all patients.

### Affymetrix GeneChip analysis

An Affymetrix Human 133U Plus 2.0 GeneChip (Affymetrix) containing 54,675 probes was used as described previously. The isolation of liver tissue RNA, amplification, hybridization, and data processing were also performed as described.^17^

### Hierarchical clustering and pathway analysis of GeneChip data

To identify differentially expressed genes between samples taken before and after l-carnitine administration, the eBayes method in the limma package was employed. Genes with a |log2-fold change| > 1 and *p* value <0.05 were considered statistically significant. Hierarchical clustering analysis was performed on the differentially expressed genes to reveal distinct expression patterns between the before and after l-carnitine administration samples. A heatmap was generated to represent visually the clustering results using the R package ComplexHeatmap. The identified differentially expressed genes were subjected to gene set enrichment analysis using the R package clusterProfiler. This analysis assessed whether predefined gene sets, such as Gene Ontology terms and Kyoto Encyclopedia of Genes and Genomes pathways, were significantly enriched among the upregulated or downregulated genes. Single-cell gene expression data of normal and cirrhotic liver (https://shiny.igc.ed.ac.uk/livercellatlas/) were utilized.^18^

### Animal studies

The generation and characterization of Ath+HFD mice were performed as described.^11^,^12^,^19^ Male C57BL/6 mice (aged 8 weeks, weighing 20–25 g) were maintained in a temperature-controlled (22±2°C) pathogen-free animal facility under a standard 12-hour light/dark cycle. Subsequently, the mice were divided randomly into 4 groups and each group was given one of the following diets for 12, 30, or 60 weeks: (I) low-fat basal diet (LFD), (II) Ath+HFD, (III) Ath+HFD supplemented with 0.5% l-carnitine, or (IV) Ath+HFD supplemented with 1% l-carnitine. The mice were killed at weeks 20 and 38 to analyze the progression of hepatic steatosis and fibrosis or at week 68 to analyze the development of hepatic tumors. The incidence of hepatic tumors and liver weight were evaluated. Blood was collected in Eppendorf tube, kept at 4°C for 30 minutes, and centrifuged at 15,000 rpm for 10 minutes at 4°C. Liver tissues were collected and snap-frozen in liquid nitrogen for proteins and in RNAlater (Thermo Fisher Scientific) for RNA extraction and analysis. A portion of the liver was fixed in 10% phosphate-buffered formalin and embedded in paraffin blocks.

All animal experiments were approved by the Ethics Committee for the Care and Use of Laboratory Animals at the Takara-Machi Campus of Kanazawa University, Japan, and were carried out in compliance with the ARRIVE guidelines 2.0. All experiments were performed in accordance with the relevant guidelines and regulations.

### Histopathology and immunohistochemical staining

Mouse liver tissues embedded in paraffin blocks in 10% formalin were stained with hematoxylin and eosin. Liver neoplasms (HCC and liver cell adenoma) were diagnosed according to previously described criteria.^11^,^12^ Hepatic fibrosis was evaluated by Azan staining using an image analysis system (BIOREVO BZ-9000; Keyence). Immunohistochemical staining was conducted by an immunoperoxidase technique with an Envision Kit (Dako). The following primary antibodies were used: rabbit polyclonal anti-PDGF receptor-beta (1:100 dilution; Cell Signaling Technology) and anti-smooth muscle actin (1:100 dilution; Santa Cruz Biotechnology).

### RNA extraction and real-time detection-PCR analysis

Total RNA was isolated from frozen liver tissue samples in RNAlater using an RNeasy Mini Kit (Qiagen) and from cell samples using a NIPPON RNA Kit (Nippon Gene). cDNA was synthesized from 100 ng total RNA using a High-capacity cDNA Reverse Transcription Kit (Thermo Fisher Scientific). Real-time detection-PCR was conducted using TaqMan Gene Expression Assay Identification. The following TaqMan probes were used: *Acta2*, *Col1a2*, *Tgfb1*, *Pdgfrb*, *Pdgfb*, *Pdgfc*, *Tnf*, *Il6*, *Il1b*, *Pparg*, *Ppara*, and *Nedd9* (neural precursor cell expressed, developmentally downregulated protein 9) (Applied Biosystems). Quantitative gene expression data were normalized to the expression levels of the housekeeping gene *GAPDH*.

### Western blotting

Whole-cell lysates from mouse liver and cultured cells were prepared and lysed in a 1× RIPA Lysis Buffer (EMD Millipore) containing complete Protease Inhibitor Cocktail and PhosSTOP (Roche Applied Science). The following primary antibodies were used: anti-NEDD9 (1:1000 dilution), anti-FAK (focal adhesion kinase) (1:1000 dilution), anti-phosphorylated (p)-FAK (Tyr397; 1:1000 dilution), anti-AKT (1:1000 dilution), anti-p-AKT (Ser473; 1:1000 dilution), and anti-GAPDH (1:1000 dilution) (Cell Signaling Technology).

### RNA interference

HepG2 cells were transfected with control (Stealth RNAi Negative Control Low GC Duplex #2; Invitrogen) or NEDD9 Stealth small-interfering RNA (Thermo Fisher Scientific) using the Lipofectamine RNAiMAX Reagent (Thermo Fisher Scientific). After 24 hours, the cells were harvested for analysis.

### Statistical analysis

Data are presented as the mean±SD and analyzed using Prism 9.4.1 (GraphPad Software, Inc.). Experiments were repeated at least 3 times. A two-tailed unpaired Student *t* test or one-way ANOVA was used to evaluate the data. Pearson correlation coefficients were used to assess the relationship. A *p* value <0.05 was considered to indicate statistical significance.

Cell culture, luciferase reporter assay, overexpression and transfection, recombinant proteins and chemicals, immunofluorescence staining, and chromatin immunoprecipitation assay are described in the Supplemental Methods, http://links.lww.com/HC9/A846.

## RESULTS

### Effects of l-carnitine administration on the pathophysiology of patients with MASH

Eleven patients with MASH (Supplemental Table S1, http://links.lww.com/HC9/A845) were administered 2 g l-carnitine daily for 10 weeks, and serum liver functions and lipid and glucose levels were compared before and after l-carnitine administration (Figure 1A). The serum levels of AST, ALT, and gamma-glutamyl transferase were significantly improved by l-carnitine administration. As for metabolic factors, triglyceride levels were significantly improved, while total cholesterol levels were not changed. The levels of fasting glucose and glycohemoglobin were not significantly changed (data not shown).

Among the 11 patients enrolled in this study, 5 were subjected to serial liver biopsies to evaluate the histological changes induced by l-carnitine. Histological examinations revealed a significant improvement in liver inflammation, NAFLD activity score, and the degree of inflammation and ballooning (Figures 1C, D). In contrast, steatosis and fibrosis stages were not improved significantly.

### Serial changes in gene expression profiles in the liver of patients with MASH after 10-week l-carnitine administration

To examine the molecular signature that was involved in the histological improvement in MASH, serial gene expression profiling using an Affymetrix gene chip (GeneChip Human Genome U133 Plus 2.0 Array) was performed with liver biopsy specimens obtained from 5 patients before and after l-carnitine administration. Pairwise comparisons before and after l-carnitine administration revealed 201 differentially expressed genes (*p* value <0.05 and fold difference >2), in which 111 genes were upregulated and 90 genes were downregulated.

**FIGURE 2:**
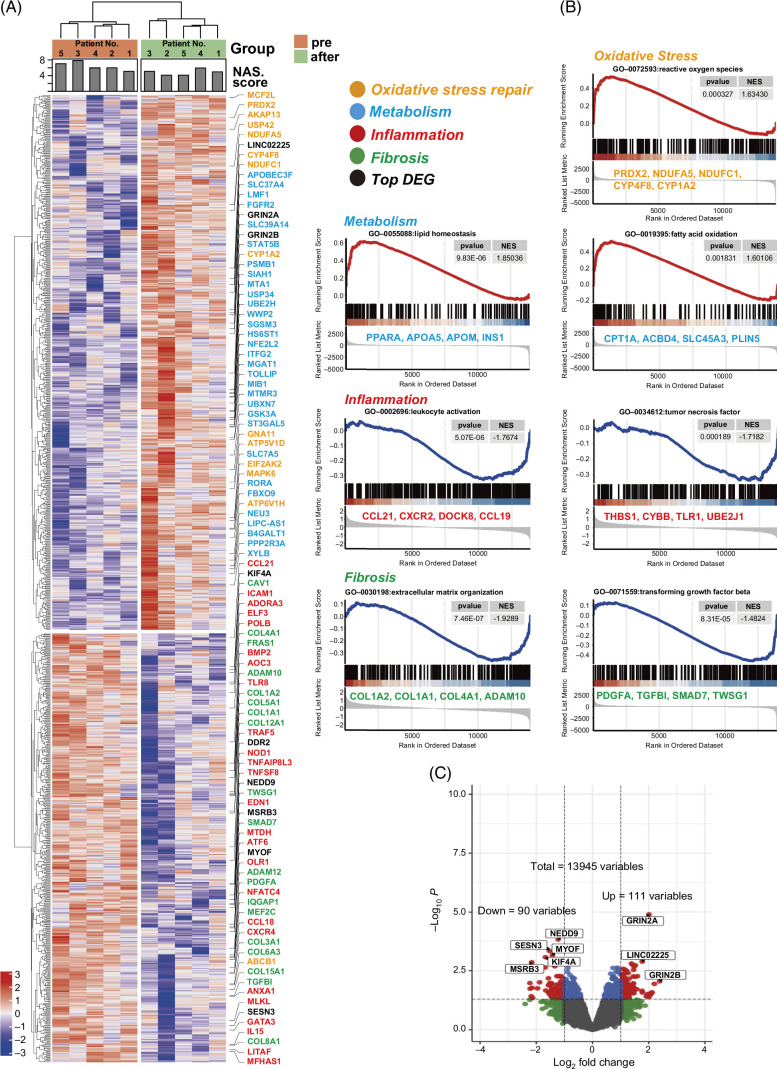
Serial changes in gene expression profiles in the liver of patients with MASH after 10-week l-carnitine administration. (A) Hierarchical clustering of 201 differentially expressed genes in the liver of paired patients with MASH before and after l-carnitine administration. Oxidative stress repair genes and metabolism-related genes were upregulated, while inflammation-related and fibrosis-related genes were downregulated. Representative genes of each category and the top differentially expressed genes are listed on the right side of the heatmap. (B) Enrichment of the pathways of the DEGs between before and after l-carnitine administration in patients with MASH. (C) Volcano plot of gene abundance based on GeneChip data. Abbreviations: DEG, differentially expressed gene; NAS, nonalcoholic fatty liver disease activity score.

A heatmap of the hierarchical clustering of the 201 differentially expressed genes is presented in Figure 2A. Overall, the degree of the upregulation and downregulation of these differentially expressed genes was well correlated with the NAFLD activity score of individual patients (Figure 2A). Gene set enrichment analysis showed reactive oxygen stress repair genes, such as peroxiredoxin 2 and ubiquinone oxidoreductase subunit A1, lipid homeostasis-related genes, such as peroxisome proliferator–activated receptor alpha and apolipoprotein A5, and fatty acid oxidation-related genes, such as carnitine palmitoyltransferase 1A and acyl-CoA binding domain-containing 4, were upregulated by l-carnitine. Conversely, leukocyte activation–related genes, such as C-C motif chemokine ligand 21 and C-X-C motif chemokine receptor 2, TNF-related genes, such as toll-like receptor 1 and thrombospondin 1, extracellular matrix–related genes, such as collagen type I alpha 2 chain and collagen type I alpha 1 chain, and TGF-β–related genes, such as PDGF subunit A and TGF-β-induced, were downregulated by l-carnitine (Figure 1B).

The results of gene set enrichment analysis derived from different gene sets (Gene Ontology vs. Kyoto Encyclopedia of Genes and Genomes) were compared (Supplemental Figure S1, http://links.lww.com/HC9/A845). The TGF-β–related pathway was the most significantly altered in the Gene Ontology database, while the AKT/FAK pathway was the most significantly altered in the Kyoto Encyclopedia of Genes and Genomes database.

Among the top 5 downregulated and top 3 upregulated genes (Figure 2C), we focused on *NEDD9*. NEDD9 is associated with the tumorigenesis of breast cancer^20^ and ovarian cancer,^21^ and the upregulation of NEDD9 in HCC is associated with epithelial-mesenchymal transition and intrahepatic metastasis^22^ and is related to poor patient prognosis.^23^ Comprehensive single-cell analysis of normal and cirrhotic liver^18^ showed that the expression of *NEDD9* was generally low in normal liver and expressed mainly in endothelial and epithelial cells (Supplemental Figures S2A, B, http://links.lww.com/HC9/A845). Interestingly, *NEDD9*-expressing hepatocytes were substantially increased in the cirrhotic liver compared with normal liver (Supplemental Figures S2C–F, http://links.lww.com/HC9/A845).

Thus, gene expression profiling showed a significant improvement in lipid metabolism, inflammation, and fibrosis signaling by the administration of l-carnitine; histological improvements were observed in liver inflammation, but not in steatosis and fibrosis (Figure 1).

### Long-term l-carnitine administration improves steatosis, inflammation, and fibrosis in Ath+HFD MASH mice

**FIGURE 3:**
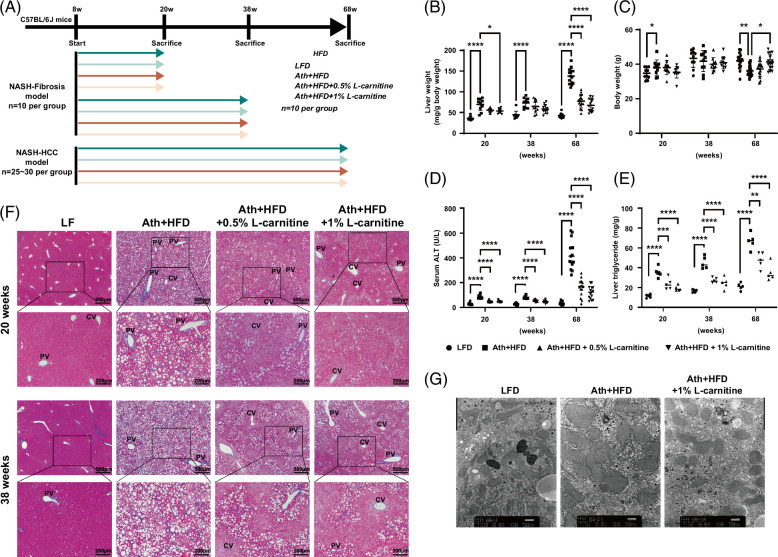
Histological improvements in a MASH mouse model by l-carnitine administration. (A) Feeding schedule of the mice. After weaning, male C57BL/6J mice were divided into 4 groups: (i) LFD, (ii) Ath+HFD, (iii) Ath+HFD supplemented with 0.5% l-carnitine, or (iv) Ath+HFD supplemented with 1% l-carnitine. (B–E) Liver weight (B), body weight (C), serum ALT (D), and liver TG (E) of mice fed the LFD, Ath+HFD, or Ath+HFD supplemented with 0.5% or 1% l-carnitine at 20, 38, and 68 weeks. (F) Hematoxylin and eosin and Azan staining of the liver of the MASH mouse model fed the LFD, Ath+HFD, or Ath+HFD supplemented with 0.5% or 1% l-carnitine at 20 and 38 weeks.(G) Electron microscopy findings in the liver of the MASH mouse model fed the LFD, Ath+HFD, or Ath+HFD supplemented with 1% l-carnitine. Scale bar: 100 μm. Data are presented as the mean±SD. *p<0.05, **p<0.01, ***p<0.001, ****p<0.0001. Abbreviations: ALT, alanine transaminase; Ath+HFD, atherogenic plus high-fat diet; CV; central vein; LFD, low-fat basal diet; MASH, metabolic dysfunction–associated steatohepatitis; PV; portal vein; TG, triglyceride.

We suspected that a 10-week l-carnitine administration may not be a long enough period to improve steatosis and fibrosis in the liver. Therefore, we evaluated the effects of long-term l-carnitine administration on the liver using an Ath+HFD MASH mouse model. An Ath+HFD containing 0.5% or 1% l-carnitine was administered to C57BL/6J mice for 20, 38, and 68 weeks, and liver histology was compared with mice receiving a non-l-carnitine–containing Ath+HFD or control LFD (Figure 3A).

At 20 and 38 weeks, liver weight was significantly increased in Ath+HFD mice compared with LFD mice, and at 68 weeks, liver weight was substantially increased in Ath+HFD mice (Figure 3B). l-carnitine administration reduced liver weight significantly. As for total body weight, Ath+HFD increased body weight at 20 weeks, but at 68 weeks, Ath+HFD decreased body weight compared with an LFD (Figure 3C). Serum ALT levels and liver triglyceride content significantly increased in Ath+HFD mice, and l-carnitine administration significantly improved these values (Figures 3D, E).

**FIGURE 4:**
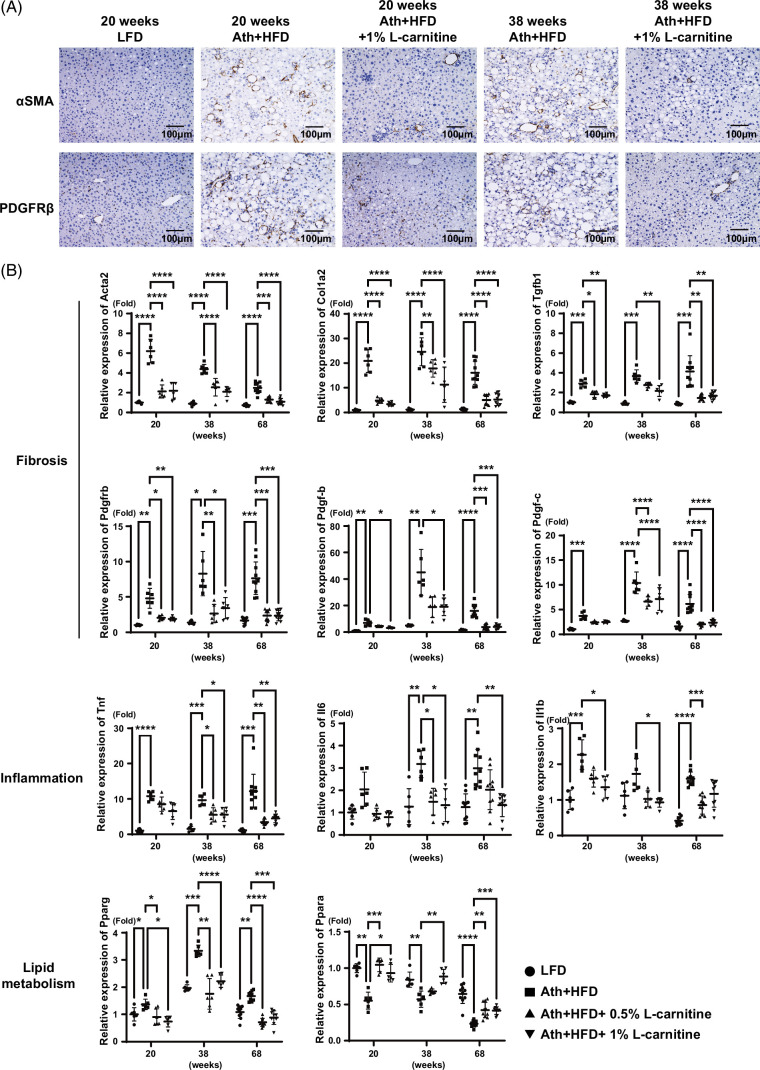
Effects of l-carnitine on the expression of fibrosis-related, inflammation-related, and steatosis-related genes in the MASH mouse model. (A) Immunohistochemical staining for αSMA and PDGFRβ in the liver of the MASH mouse model fed the LFD, Ath+HFD, or Ath+HFD supplemented with 1% l-carnitine at 20 and 38 weeks. (B) Relative expression of *Acta2*, *Col1a2*, *Tgfb1*, *Pdgfrb*, *Pdgf-b*, *Pdgf-c*, *Tnf*, *Il6*, *Il1b*, *Pparg*, and *Ppara* mRNA in the liver of the MASH mouse model fed the LFD, Ath+HFD, or Ath+HFD supplemented with 0.5% or 1% l-carnitine at 20, 38, and 68 weeks. Scale bar: 100 μm. Data are presented as the mean±SD. *p<0.05, **p<0.01, ***p<0.001, ****p<0.0001. Abbreviations: αSMA, alpha-smooth muscle actin; Ath+HFD, atherogenic plus high-fat diet; LFD, low-fat basal diet; MASH, metabolic dysfunction–associated steatohepatitis; PDGFRβ, platelet-derived growth factor receptor-beta.

Histological examination revealed that Ath+HFD substantially increased liver steatosis, inflammation, and fibrosis at 20 and 38 weeks, while l-carnitine improved liver histology in a dose-dependent manner (Figure 3F). Interestingly, l-carnitine predominantly rescued pericentral (zone 3) lesions (Figure 3F). Electron microscopy showed the presence of swollen and broken mitochondria in the liver of Ath+HFD mice, while l-carnitine rescued the abnormal mitochondria, reflecting the reduction of oxidative stress (Figure 3G). Immunohistochemical staining of α-smooth muscle actin and PDGF receptor-β demonstrated a significant improvement in profibrosis signaling by l-carnitine (Figure 4A).

Correlated with these findings, quantitative real-time detection-PCR analysis showed that the mRNA expression of profibrosis genes such as *Acta2*, *Col1a2*, *Tgfb1*, *Pdgfrb*, *Pdgf-b*, and *Pdgf-c* was significantly upregulated in the liver of Ath+HFD mice, and l-carnitine significantly suppressed the expression of these genes at 20, 38, and 68 weeks (Figure 4B). Similarly, for inflammation-related genes, *Tnfa*, *Il6*, *and Il1b* mRNA expression was significantly upregulated in the liver of Ath+HFD mice, and l-carnitine significantly suppressed the expression of these genes at 20, 38, and 68 weeks (Figure 4B). For lipid metabolism–related genes, *Pparg* mRNA expression was significantly upregulated in the liver of Ath+HFD mice, and l-carnitine effectively suppressed its expression, while *Ppara* expression was significantly downregulated in the liver of Ath+HFD mice, and l-carnitine effectively rescued its expression (Figure 4B). These results were concordant with those of recent reports using hepatocyte-specific gene knockout mice showing that *Pparg* accelerates MASH^24^ and *Ppara* has protective roles against MASH.^25^

### l-Carnitine suppresses the development of liver tumors in Ath+HFD MASH mice

**FIGURE 5:**
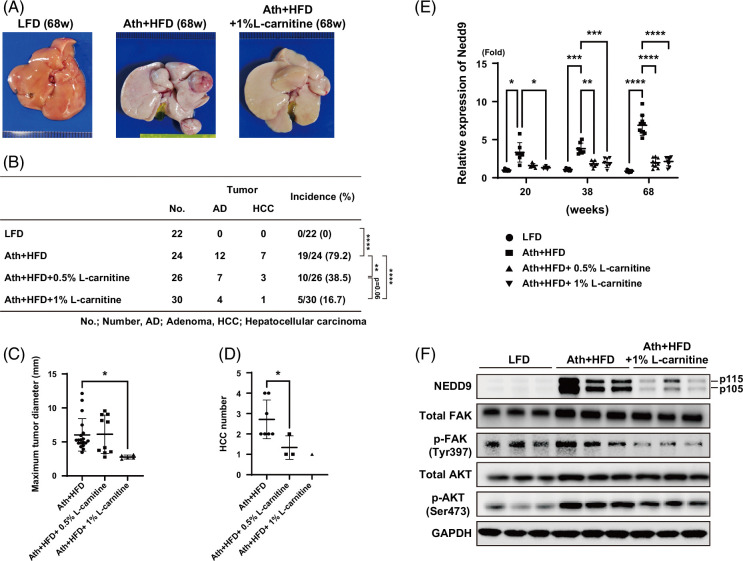
Effects of l-carnitine on the development of liver tumors in the MASH mouse model. (A) Macroscopic findings and (B) the incidence of hepatic tumors (adenoma or HCC) in the liver of the MASH mouse model fed the LFD, Ath+HFD, or Ath+HFD supplemented with 0.5% or 1% l-carnitine at 68 weeks. (C) Maximum tumor diameter and (D) tumor number in the liver of the MASH mouse model fed the LFD, Ath+HFD, or Ath+HFD supplemented with 0.5% or 1% l-carnitine at 68 weeks. (E) Relative expression of *Nedd9* mRNA in the liver of the MASH mouse model fed the LFD, Ath+HFD, or Ath+HFD supplemented with 0.5% or 1% l-carnitine at 20, 38, and 68 weeks. (F) Western blotting of NEDD9, FAK, p-FAK, AKT, p-AKT, and GAPDH in the liver of the MASH mouse model fed the LFD, Ath+HFD, or Ath+HFD supplemented with 1% l-carnitine at 68 weeks. Data are presented as the mean±SD. *p<0.05, **p<0.01, ***p<0.001, ****p<0.0001. Abbreviations: Ath+HFD, atherogenic plus high-fat diet; FAK, focal adhesion kinase; LFD, low-fat basal diet; MASH, metabolic dysfunction–associated steatohepatitis; NEDD9, neural precursor cell expressed, developmentally downregulated protein 9.

Ath+HFD MASH model mice developed liver tumors at 68 weeks (Figure 5A); 79.2% of Ath+HFD mice developed liver tumors (12 adenoma and 7 HCC out of 24 mice), while no tumors were observed in LFD mice (*p*<0.0001) (Figure 5B). The administration of 0.5% l-carnitine significantly reduced the incidence of liver tumors in Ath+HFD mice (38.5%; 7 adenoma and 3 HCC out of 26 mice; *p*<0.001), and 1% l-carnitine also reduced the incidence of liver tumors (16.7%; 4 adenoma and 1 HCC out of 30 mice; *p*<0.0001), although the difference between 0.5% and 1% l-carnitine did not reach statistical significance (*p*=0.06). Maximum tumor diameter and the incidence of HCC were significantly reduced by l-carnitine administration (Figures 5C, D).

As *NEDD9* was one of the top 5 genes downregulated by l-carnitine administration in patient liver samples (Figure 2C), we examined *Nedd9* expression in Ath+HFD mice (Figure 5E). *Nedd9* expression was significantly upregulated in the liver of Ath+HFD mice compared with LFD mice and its expression was gradually increased over the period of Ath+HFD feeding (~7-fold increase at 68 weeks). l-Carnitine administration significantly repressed *Nedd9* expression (Figure 5E).

Western blotting of whole liver lysates from Ath+HFD mice confirmed the presence of the 2 differentially phosphorylated forms of NEDD9 (p115 and p105, respectively), as described.^26^ NEDD9 expression was substantially increased in the liver of Ath+HFD mice and was repressed by l-carnitine (Figure 5F). NEDD9 is a noncatalytic C10 regulator of kinase-associated substrate family scaffolding protein that mediates the function of many oncogenic proteins.^27^ NEDD9 protein has a conserved NH_2_-terminal Src homology 3 domain that binds to proteins containing polyproline motifs such as FAK.^27^ NEDD9 and FAK regulate diverse cellular processes, including growth factor signaling, cell cycle progression, cell survival, cell motility, and angiogenesis, through their kinase-dependent and kinase-independent scaffolding functions.^28^ p-FAK levels were increased in the liver of Ath+HFD mice and repressed by l-carnitine (Figure 5F). Similarly, p-AKT levels were increased in the liver of Ath+HFD mice, while they were repressed by l-carnitine administration (Figure 5F).

### l-Carnitine represses NEDD9/FAK/AKT signaling in hepatocytes

**FIGURE 6:**
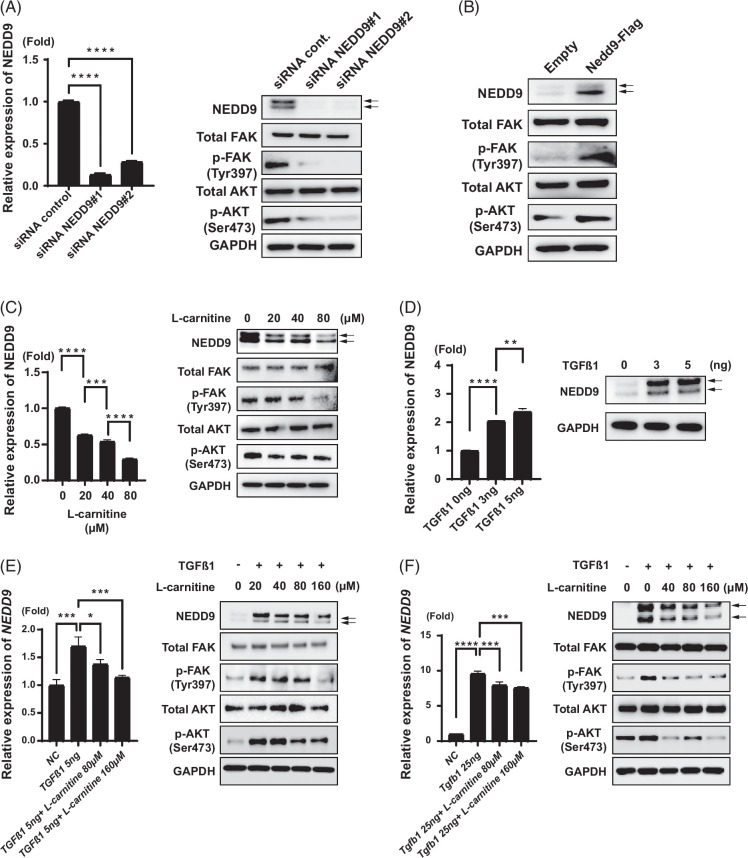
l-Carnitine inhibits NEDD9/FAK/AKT signaling in HepG2 cells and mouse primary hepatocytes. (A) siRNA targeting *NEDD9* efficiently suppressed NEDD9 mRNA (left) and protein expression and p-FAK and p-AKT (right) levels in HepG2 cells. (B) Overexpression of FLAG-tagged NEDD9 increased p-FAK and p-AKT levels in HepG2 cells. Empty was vehicle vector alone. (C) l-Carnitine decreased NEDD9 mRNA (left) and protein (right) expression in a dose-dependent manner in HepG2 cells. p-FAK and p-AKT levels were subsequently repressed. (D) Recombinant human TGF-β1 increased NEDD9 mRNA and protein expression in HepG2 cells after 24-hour stimulation. (E, F) l-Carnitine decreased the TGF-β1–induced expression of NEDD9 and p-FAK and p-AKT levels in HepG2 (E) and mouse primary hepatocytes (F). Data are presented as the mean±SD. *p<0.05, **p<0.01, ***p<0.001, ****p<0.0001. Abbreviations: FAK, focal adhesion kinase; NEDD9, neural precursor cell expressed, developmentally downregulated protein 9; siRNA, small-interfering ribonucleic acid.

We examined whether l-carnitine could repress NEDD9/FAK/AKT signaling in hepatocytes. We first confirmed the association of NEDD9, FAK, and AKT signaling in HepG2 cells, a hepatoblastoma cell line. Knocking down NEDD9 expression by small-interfering RNA efficiently reduced the levels of p-FAK (Tyr397) and p-AKT (Ser473) in HepG2 cells (Figure 6A). Conversely, NEDD9 overexpression increased p-FAK and p-AKT levels in HepG2 cells (Figure 6B). Thus, NEDD9/FAK/AKT signaling was active in HepG2 cells. Interestingly, we found that l-carnitine treatment reduced NEDD9 expression at the mRNA and protein levels in HepG2 cells (Figure 6C). Following the repression of NEDD9 expression, p-FAK and p-AKT levels were reduced (Figure 6C).

TGF-β1 treatment significantly increased NEDD9 expression at the mRNA and protein levels in HepG2 cells (Figure 6D). We examined whether l-carnitine could repress the TGF-β1–induced expression of NEDD9 in HepG2 cells (Figure 6E) and mouse primary hepatocytes (Figure 6F). Although a high concentration of l-carnitine was required to reduce TGF-β1–induced NEDD9 expression in HepG2 cells (160 μM), a low concentration of l-carnitine (from 40 μM) could repress TGF-β1–induced NEDD9 expression in mouse primary hepatocytes. Following the repression of NEDD9, p-FAK, and p-AKT levels were reduced in HepG2 cells and mouse primary hepatocytes (Figures 6E, F).

### l-Carnitine reduces NEDD9 expression by regulating the expression of early growth response 1

**FIGURE 7:**
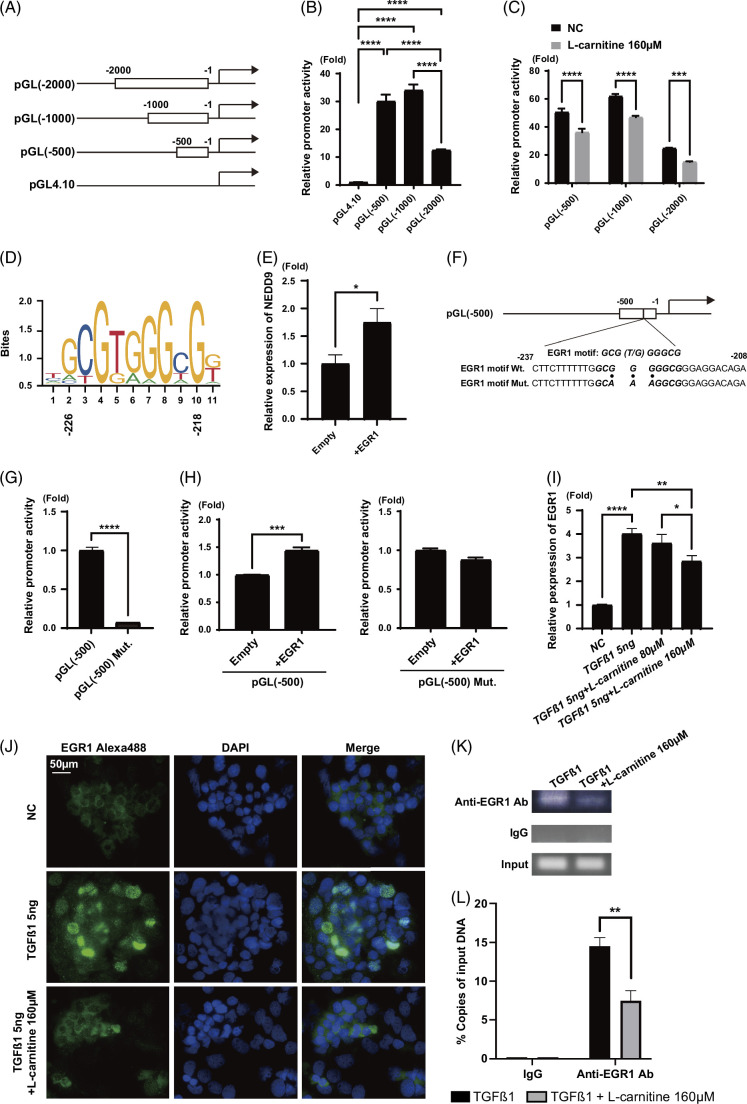
l-Carnitine regulates NEDD9 expression possibly through the stress-induced transcription factor EGR1. (A) Construction of NEDD9 promoter assay constructs. pGL(−2000): including −2,000 to 0 bases relative to the transcription initiation site of NEDD9 fused to a firefly luciferase gene. pGL(−1000) and pGL(−500): reporter constructs containing serial deletions of the putative promoter region. (B) Promoter activity of a series of NEDD9 promoter constructs. (C) Effect of l-carnitine on the activity of NEDD9 promoter constructs. (D) Putative upstream transcription factors of NEDD9 and the EGR1-binding site were predicted using the JASPAR database (https://jaspar.genereg.net/). (E) Transfection of a full-length EGR1 expression vector increased *NEDD9* mRNA expression in HepG2 cells. (F) Mutations introduced into the EGR1-binding site. (G) Promoter activity was assessed in HepG2 cells transfected with pGL(−500) or pGL(−500) (mutated [Mut]). (H) Promoter activity was assessed in HepG2 cells cotransfected with pGL(−500) or pGL(−500) (Mut) along with an EGR1 overexpression vector or empty vector. (I) l-Carnitine decreased the TGF-β1–induced expression of *EGR1* mRNA. (J) l-Carnitine inhibited the nuclear translocation of EGR1 induced by TGF-β1 in HepG2 cells. (K) A chromatin immunoprecipitation assay was conducted in HepG2 cells treated with TGF-β1, with and without l-carnitine. l-Carnitine decreased the pull down of EGR1-binding chromatin DNA that was subsequently precipitated using an anti-EGR1 antibody (Ab) but not control IgG. (L) Quantitative measurement of ChIP-precipitated DNA (Supplemental Figure S3, http://links.lww.com/HC9/A845). Scale bar: 50 μm. Data are presented as the mean±SD. *p<0.05, **p<0.01, ***p<0.001, ****p<0.0001. Abbreviations: ChIP, chromatin immunoprecipitation; EGR1, early growth response 1; IgG, Immunogloblin G; NEDD9, neural precursor cell expressed, developmentally downregulated protein 9.

To examine the molecular mechanisms by which l-carnitine reduced NEDD9 expression, we examined the promoter region of NEDD9. Upstream regions (−500, −1000, and −2000 bases) from the transcription start site were cloned into a luciferase reporter vector (pGL[−500], pGL[−1000], and pGL[−2000], respectively) (Figure 7A). Promoter activity was observed from all 3 constructs in HepG2 cells, although pGL(−2000) showed less activity than pGL(−500) and pGL(−1000) (Figure 7B). l-Carnitine treatment significantly decreased promoter activity in all 3 constructs (Figure 7C), suggesting that a regulatory region of l-carnitine is present in pGL(−500). Therefore, we searched the primary sequence up to 500 bases upstream from the transcription start site and identified a putative early growth response 1 (EGR1)-binding motif at −218 to −226 bases (GCGT/GGGGCG) (Figure 7D). We confirmed that EGR1 overexpression increased NEDD9 expression in HepG2 cells (Figure 7E). Interestingly, when mutations were introduced into the EGR1-binding motif, the promoter activity of pGL(−500) was substantially decreased (Figures 7F, G), suggesting that EGR1 was required for the basal promoter activity of NEDD9. EGR1 overexpression significantly increased the promoter activity of pGL(−500), while it failed to increase the promoter activity of pGL(−500) with EGR1-binding motif mutations (Figure 7H).

We next examined the relationship between the expression of TGF-β1 and EGR1. TGF-β1 treatment significantly upregulated *EGR1* mRNA expression in HepG2 cells (Figure 7I). Interestingly, 160 μM l-carnitine significantly reduced *EGR1* expression (Figure 7I). Immunofluorescence staining showed that TGF-β1 increased EGR1 expression; interestingly, the nuclear accumulation of EGR1 was observed. l-Carnitine reduced the expression and nuclear accumulation of EGR1 (Figure 7J).

A chromatin immunoprecipitation assay showed that l-carnitine reduced the amount of a chromatin region in the promoter of NEDD9 (from −193 to −266) containing the EGR1-binding motif (−218 to −226) that was pulled down (Figure 7K). Quantitative measurement of chromatin immunoprecipitation–precipitated DNA revealed that a significantly lower amount of DNA was pulled down from l-carnitine–treated cells (Figure 7L) (Supplemental Figure S3, http://links.lww.com/HC9/A845).

These results showed that l-carnitine repressed NEDD9 expression through the repression of *EGR1* transcription and inhibition of the nuclear translocation of EGR1.

## DISCUSSION

MASH has emerged as a significant global health concern, progressively supplanting viral hepatitis as the primary cause of HCC. Despite its increasing prevalence, the prevention of MASH-derived HCC is a substantial challenge due to the unique molecular and metabolic features of MASH.

l-Carnitine, a vital human nutrient, is traditionally recognized for its role in transferring long-chain fatty acids to the mitochondrial matrix and subsequent promotion of energy metabolism and ATP production by activating the β-oxidation of fatty acids. Recent research has highlighted its potential in ameliorating various metabolic disorders such as hyperlipidemia,^3^ hyperglycemia,^4^ and obesity.^2^

Various clinical studies have been applied to MASLD/MASH, and meta-analyses suggest that l-carnitine treatment can significantly improve serum ALT and AST levels as well as NAFLD activity scores in patients with MASH.^10^ However, the molecular events induced by l-carnitine in the liver of patients with MASH have not been clarified comprehensively. In this study, we demonstrated the changes in the hepatic gene expression profiles of patients with MASH associated with l-carnitine administration for the first time. Although the serum levels of ALT/AST and histological inflammation score (NAFLD activity score) were significantly improved in the liver of patients with MASH, the degree of steatosis and fibrosis stage were not improved (Figure 1 and Supplemental Table S1, http://links.lww.com/HC9/A845). This might be due to the relatively short period of l-carnitine administration, as the hepatic gene expression profiles showed a substantial improvement in inflammation-related and profibrotic-related genes along with the recovery of lipid metabolism–related genes (Figures 2 and 3). It was noteworthy that l-carnitine showed strong anti-inflammatory effects rather than effects on lipid metabolism (Figures 1 and 2 and Supplemental Figure S1, http://links.lww.com/HC9/A845). Previous reports have demonstrated that l-carnitine is involved in the antioxidant effect against reactive oxygen species in hepatocytes,^29^ proximal tubule epithelial cells,^30^ lens epithelial cells,^31^ ovary cells,^32^ and mouse embryos.^33^ l-Carnitine effectively diminishes H_2_O_2_-induced cell apoptosis^33^ by stabilizing antioxidant proteins such as superoxidase dismutase.^34^ Fundamentally, l-carnitine increases fatty acid transfer into mitochondria and stimulates fatty acid β-oxidation, which potentially generates reactive oxygen species; therefore, it could be assumed that l-carnitine might also be involved in reactive oxygen species removal pathways. That might be one of the reasons why the anti-inflammatory effects of l-carnitine were prominent in this clinical study.

To evaluate the long-term effect of l-carnitine on MASH, we took advantage of a MASH-HCC mouse model, as reported.^11^,^12^ Although l-carnitine prevents hepatocarcinogenesis in a Long-Evans Cinnamon rat HCC model^35^ or STAM-HCC mouse model,^7^ these models are different from human MASH in terms of genetic modification or lack of insulin resistance.^36^ Ath+HFD induces dyslipidemia, lipid peroxidation, and stellate cell activation in the liver and finally causes precirrhotic steatohepatitis after 24 weeks accompanied by cellular ballooning and hepatic insulin resistance.^19^ Furthermore, mice develop liver tumors at a high frequency at 68 weeks.^11^,^12^

We found that long-term l-carnitine administration substantially ameliorated the liver histology observed in the Ath+HFD MASH mouse model. Liver inflammation, steatosis, and fibrosis were significantly improved (Figures 3 and 4), and at 68 weeks, l-carnitine significantly reduced the incidence of liver tumors (Figure 5). This is the first report showing that l-carnitine prevents hepatocarcinogenesis in a MASH mouse model. Interestingly, l-carnitine predominantly rescued zone 3 lesions (Figure 3F). Because β-oxidation of fatty acids is more active in zone 1, l-carnitine should rescue lesions in zone 1 rather than in zone 3. The reasons why l-carnitine rescued lesions in zone 3 rather than in zone 1 in this study could not be determined. However, the antioxidant redox signal was found to be more active in zone 3,^37^ and the redox signal might be induced by l-carnitine. The second possibility is that a regeneration of the zone 3 area might occur, given that l-carnitine is reported to enhance liver regeneration.^38^ Although Axin-2–positive progenitor cells in zone 3 had limited self-renewal capacity, continuous cell death with HFD in MUP (major mouse urinary protein) mice potentiated zone 3 cell expansion.^39^

As an l-carnitine target gene, we focused on *NEDD9*, which was among the top 5 genes downregulated by l-carnitine in the liver of patients with MASH (Figure 2). NEDD9 is a C10 regulator of kinase-associated substrate family scaffolding protein that mediates the function of many oncogenic proteins.^27^ NEDD9 promotes oncogenic signaling in the development of mammary tumors^20^ and ovarian cancer^21^ and could be an oncogenic driver in mouse liver tumor models.^40^ The *NEDD9* mRNA and protein expression levels in HCC tissues were significantly higher than those in matched adjacent nontumor hepatic tissues, and patients with high NEDD9 expression levels exhibited poorer recurrence-free and overall survival than those with low NEDD9 expression.^23^ NEDD9 associated with FAK, which is a nonreceptor tyrosine kinase that is overexpressed and activated in many cancer types. NEDD9 and FAK regulate diverse cellular processes, including growth factor signaling, cell cycle progression, cell survival, cell motility, and angiogenesis.^28^

We found that *Nedd9* expression was increased in our mouse Ath+HFD MASH model and its expression was gradually increased by up to ~7-fold at 68 weeks (Figure 5E). The expression of *NEDD9* is generally low in normal liver. Single-cell analysis using a public database showed that *NEDD9* was mainly expressed in endothelial and epithelial cells in normal liver (Supplemental Figures S2A, B, http://links.lww.com/HC9/A845).^18^ Interestingly, *NEDD9*-expressing hepatocytes were substantially increased in liver cirrhosis, but its expression was not changed in endothelial cells or cholangiocytes (Supplemental Figures S2C–F, http://links.lww.com/HC9/A845). Therefore, the upregulation of *Nedd9* expression in the liver might be related to its increased levels in hepatocytes, which are potentially linked to the development of liver tumors.

l-Carnitine effectively reduced *Nedd9* expression in the liver of Ath+HFD MASH mice (Figure 5E), and in concordance with NEDD9 expression, p-FAK and p-AKT levels were increased in this model and reduced by l-carnitine (Figure 5F). The correlation between NEDD9 and p-FAK and p-AKT was more clearly shown in a hepatoblastoma-derived cell line (HepG2 cells) (Figures 6A, B). Interestingly, we found that l-carnitine directly repressed NEDD9 expression in HepG2 cells (Figure 6C). We also showed that l-carnitine could reduce TGF-β1–induced NEDD9 expression in HepG2 cells (Figure 6E) and mouse primary hepatocytes (Figure 6F), together with p-FAK and p-AKT levels.

We searched the promoter region of NEDD9 and identified a putative EGR1-binding motif (Figure 7). Mutational analysis of the EGR1-binding motif showed the functional importance of EGR1 on the basal expression of NEDD9. EGR1 is an immediate early transcriptional factor that acts as a coordinator of the complex response to stress and is induced during liver injury. The association between EGR1 and MASLD/MASH has been reported recently. *Egr1* is upregulated by insulin in hepatoma cells.^41^ *Egr1*-deficient mice fed an HFD are less susceptible to diet-induced obesity and obesity-associated disorders such as insulin resistance, hyperinsulinemia, hyperlipidemia, and fatty liver.^42^ Moreover, EGR1 regulates the expression of many cholesterol biosynthetic genes.^43^ Therefore, the increased expression of NEDD9 through EGR1 observed in this study would be applicable to other MASH models. In fact, *EGR1* expression was well correlated with the progression or regression of liver fibrosis in our cohort of serial biopsied MASH liver samples (data not shown).^14^ In addition, we previously reported that EGR1 might be a key regulator of the development of HCC in patients with chronic hepatitis C.^44^

**FIGURE 8:**
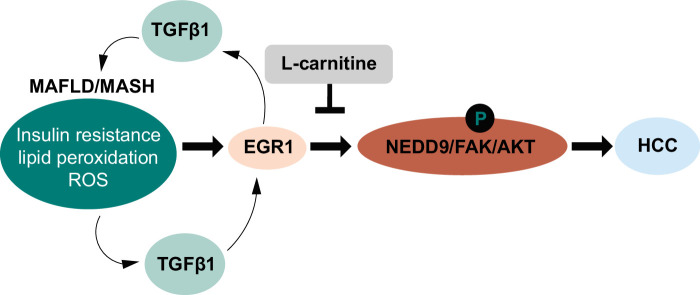
Schematic representation of how l-carnitine prevents the progression of MASH and the development of liver tumors. Insulin resistance, lipid peroxidation, and reactive oxygen species generation in MASH liver induce the expression of the stress-induced transcriptional factor EGR1. TGF-β1 accelerates the pathophysiology of MASH by acting as a positive feedback regulator between MASH and EGR1, which potentially activates the NEDD9/FAK/AKT oncogenic signaling pathway. l-carnitine could ameliorate EGR1-mediated NEDD9 expression by reducing ROS generation, improving lipid metabolism, and activating anti-inflammatory activity. Abbreviations: EGR1, early growth response 1; FAK, focal adhesion kinase; MASH, metabolic dysfunction–associated steatohepatitis; NEDD9, neural precursor cell expressed, developmentally downregulated protein 9; ROS, reactive oxygen species.

We showed that TGF-β1 treatment significantly upregulated *EGR1* mRNA expression in HepG2 cells (Figure 7I). Conversely, EGR1 reportedly stimulates TGF-β1 expression through binding to the EGR1-binding site in its promoter^45^; therefore, there might be a positive feedback loop between TGF-β1 and EGR1. Interestingly, l-carnitine significantly reduced the TGF-β1–induced expression of *EGR1* mRNA (Figure 7I), and further, it decreased the nuclear accumulation and binding to the *NEDD9* promoter of EGR1 (Figures 7J–L). Although the effect of l-carnitine on EGR1 expression should be clarified in more detail, these results revealed the direct effect of l-carnitine on NEDD9 expression through EGR1 (Figure 8).

This study has some limitations. First, l-carnitine administration for 10 weeks resolved liver inflammation but did not improve steatosis and fibrosis stage in patients with MASH. An extended period of l-carnitine administration would be required to evaluate the real effects of l-carnitine in preventing steatosis, fibrosis, and the occurrence of liver tumors. Second, although l-carnitine reduced the incidence of liver tumors in the Ath+HFD MASH model, the effects of l-carnitine on other MASH tumor models such as the gubra amylin NASH diet-induced obese MASH-HCC (gubra amylin NASH diet-induced obese-MASH-HCC) model^46^ still need to be evaluated. The Ath+HFD MASH model showed reduced body weight at a higher age (68 wk) (Figure 3C), whereas the gubra amylin NASH diet-induced obese-MASH model maintained body weight at a higher age (72 wk).^46^

In conclusion, we demonstrated that l-carnitine potentially improved the pathophysiology of MASLD/MASH and inhibited the subsequent development of HCC. We showed that l-carnitine directly inhibited one of the NALFD/MASH-mediated oncogenic pathways, EGR1/NEDD9/FAK/AKT. Further clinical trials with a longer duration of l-carnitine administration should be conducted to demonstrate the proof of concept for the clinical usage of l-carnitine to prevent the progression of MASLD/MASH to HCC.

## Supplementary Material

## Data Availability

The microarray data for transcriptome analysis has been deposited in the GEO database (www.ncbi.nlm.nih.gov/geo/) with the dataset identifier GSE261938.

## References

[1] Rinella ME Lazarus JV Ratziu V Francque SM Sanyal AJ Kanwal F. A multi-society Delphi consensus statement on new fatty liver disease nomenclature. Ann Hepatol. 2023;78:101133. 10.1016/j.aohep.2023.101133 37364816

[2] Talenezhad N Mohammadi M Ramezani-Jolfaie N Mozaffari-Khosravi H Salehi-Abargouei A. Effects of l-carnitine supplementation on weight loss and body composition: A systematic review and meta-analysis of 37 randomized controlled clinical trials with dose-response analysis. Clin Nutr ESPEN. 2020;37:9–23. 32359762 10.1016/j.clnesp.2020.03.008

[3] Musazadeh V Alinejad H Esfahani NK Kavyani Z Keramati M Roshanravan N. The effect of L-carnitine supplementation on lipid profile in adults: An umbrella meta-analysis on interventional meta-analyses. Front Nutr. 2023;10:1214734. 37727632 10.3389/fnut.2023.1214734 PMC10506516

[4] Zamani M Pahlavani N Nikbaf-Shandiz M Rasaei N Ghaffarian-Ensaf R Asbaghi O. The effects of L-carnitine supplementation on glycemic markers in adults: A systematic review and dose-response meta-analysis. Front Nutr. 2022;9:1082097. 36704801 10.3389/fnut.2022.1082097 PMC9871499

[5] Dahash BA Sankararaman S. Carnitine Deficiency. StatPearls; 2023. 32644467

[6] Andrieu-Abadie N Jaffrezou JP Hatem S Laurent G Levade T Mercadier JJ. L-carnitine prevents doxorubicin-induced apoptosis of cardiac myocytes: Role of inhibition of ceramide generation. FASEB J. 1999;13:1501–1510. 10463940 10.1096/fasebj.13.12.1501

[7] Ishikawa H Takaki A Tsuzaki R Yasunaka T Koike K Shimomura Y. L-carnitine prevents progression of non-alcoholic steatohepatitis in a mouse model with upregulation of mitochondrial pathway. PLoS One. 2014;9:e100627. 24983359 10.1371/journal.pone.0100627 PMC4077577

[8] Kon K Ikejima K Morinaga M Kusama H Arai K Aoyama T. L-carnitine prevents metabolic steatohepatitis in obese diabetic KK-A(y) mice. Hepatol Res. 2017;47:E44–E54. 27062266 10.1111/hepr.12720

[9] Mollica G Senesi P Codella R Vacante F Montesano A Luzi L. L-carnitine supplementation attenuates NAFLD progression and cardiac dysfunction in a mouse model fed with methionine and choline-deficient diet. Dig Liver Dis. 2020;52:314–323. 31607566 10.1016/j.dld.2019.09.002

[10] Liu A Cai Y Yuan Y Liu M Zhang Z Xu Y. Efficacy and safety of carnitine supplementation on NAFLD: A systematic review and meta-analysis. Syst Rev. 2023;12:74. 37120548 10.1186/s13643-023-02238-w PMC10148537

[11] Okada H Takabatake R Honda M Takegoshi K Yamashita T Nakamura M. Peretinoin, an acyclic retinoid, suppresses steatohepatitis and tumorigenesis by activating autophagy in mice fed an atherogenic high-fat diet. Oncotarget. 2017;8:39978–39993. 28591717 10.18632/oncotarget.18116 PMC5522259

[12] Takegoshi K Honda M Okada H Takabatake R Matsuzawa-Nagata N Campbell JS. Branched-chain amino acids prevent hepatic fibrosis and development of hepatocellular carcinoma in a non-alcoholic steatohepatitis mouse model. Oncotarget. 2017;8:18191–18205. 28212548 10.18632/oncotarget.15304 PMC5392319

[13] Takeshita Y Honda M Harada K Kita Y Takata N Tsujiguchi H. Comparison of tofogliflozin and glimepiride effects on nonalcoholic fatty liver disease in participants with type 2 diabetes: A randomized, 48-week, open-label, active-controlled trial. Diabetes Care. 2022;45:2064–2075. 35894933 10.2337/dc21-2049 PMC9472500

[14] Sako S Takeshita Y Takayama H Goto H Nakano Y Ando H. Trajectories of liver fibrosis and gene expression profiles in nonalcoholic fatty liver disease associated with diabetes. Diabetes. 2023;72:1297–1306. 37343270 10.2337/db22-0933

[15] Brunt EM Janney CG Di Bisceglie AM Neuschwander-Tetri BA Bacon BR. Nonalcoholic steatohepatitis: A proposal for grading and staging the histological lesions. Am J Gastroenterol. 1999;94:2467–2474. 10484010 10.1111/j.1572-0241.1999.01377.x

[16] Kleiner DE Brunt EM Van Natta M Behling C Contos MJ Cummings OW. Design and validation of a histological scoring system for nonalcoholic fatty liver disease. Hepatology. 2005;41:1313–1321. 15915461 10.1002/hep.20701

[17] Honda M Sakai A Yamashita T Nakamoto Y Mizukoshi E Sakai Y. Hepatic ISG expression is associated with genetic variation in interleukin 28B and the outcome of IFN therapy for chronic hepatitis C. Gastroenterology. 2010;139:499–509. 20434452 10.1053/j.gastro.2010.04.049

[18] Ramachandran P Dobie R Wilson-Kanamori JR Dora EF Henderson BEP Luu NT. Resolving the fibrotic niche of human liver cirrhosis at single-cell level. Nature. 2019;575:512–518. 31597160 10.1038/s41586-019-1631-3 PMC6876711

[19] Matsuzawa N Takamura T Kurita S Misu H Ota T Ando H. Lipid-induced oxidative stress causes steatohepatitis in mice fed an atherogenic diet. Hepatology. 2007;46:1392–1403. 17929294 10.1002/hep.21874

[20] Izumchenko E Singh MK Plotnikova OV Tikhmyanova N Little JL Serebriiskii IG. NEDD9 promotes oncogenic signaling in mammary tumor development. Cancer Res. 2009;69:7198–7206. 19738060 10.1158/0008-5472.CAN-09-0795 PMC2758619

[21] Gabbasov R Xiao F Howe CG Bickel LE O’Brien SW Benrubi D. NEDD9 promotes oncogenic signaling, a stem/mesenchymal gene signature, and aggressive ovarian cancer growth in mice. Oncogene. 2018;37:4854–4870. 29773902 10.1038/s41388-018-0296-y PMC6119087

[22] Zhou S Xu M Shen J Liu X Chen M Cai X. Overexpression of NEDD9 promotes cell invasion and metastasis in hepatocellular carcinoma. Clin Res Hepatol Gastroenterol. 2017;41:677–686. 28578938 10.1016/j.clinre.2017.04.011

[23] Lu P Wang ZP Dang Z Zheng ZG Li X Zhou L. Expression of NEDD9 in hepatocellular carcinoma and its clinical significance. Oncol Rep. 2015;33:2375–2383. 25812772 10.3892/or.2015.3863

[24] Lee SM Pusec CM Norris GH De Jesus A Diaz-Ruiz A Muratalla J. Hepatocyte-specific loss of PPARgamma protects mice from NASH and increases the therapeutic effects of rosiglitazone in the liver. Cell Mol Gastroenterol Hepatol. 2021;11:1291–1311. 33444819 10.1016/j.jcmgh.2021.01.003 PMC8005819

[25] Regnier M Polizzi A Smati S Lukowicz C Fougerat A Lippi Y. Hepatocyte-specific deletion of Pparalpha promotes NAFLD in the context of obesity. Sci Rep. 2020;10:6489. 32300166 10.1038/s41598-020-63579-3 PMC7162950

[26] Liu X Elia AE Law SF Golemis EA Farley J Wang T. A novel ability of Smad3 to regulate proteasomal degradation of a Cas family member HEF1. EMBO J. 2000;19:6759–6769. 11118211 10.1093/emboj/19.24.6759 PMC305889

[27] Singh MK Dadke D Nicolas E Serebriiskii IG Apostolou S Canutescu A. A novel Cas family member, HEPL, regulates FAK and cell spreading. Mol Biol Cell. 2008;19:1627–1636. 18256281 10.1091/mbc.E07-09-0953 PMC2291417

[28] Chuang HH Zhen YY Tsai YC Chuang CH Hsiao M Huang MS. FAK in cancer: From mechanisms to therapeutic strategies. Int J Mol Sci. 2022;23:1726. 35163650 10.3390/ijms23031726 PMC8836199

[29] Li JL Wang QY Luan HY Kang ZC Wang CB. Effects of L-carnitine against oxidative stress in human hepatocytes: Involvement of peroxisome proliferator-activated receptor alpha. J Biomed Sci. 2012;19:32. 22435679 10.1186/1423-0127-19-32 PMC3338374

[30] Ye J Li J Yu Y Wei Q Deng W Yu L. L-carnitine attenuates oxidant injury in HK-2 cells via ROS-mitochondria pathway. Regul Pept. 2010;161:58–66. 20093144 10.1016/j.regpep.2009.12.024

[31] Li X Meng F Li H Hua X Wu L Yuan X. L-carnitine alleviates oxidative stress‑related damage via MAPK signaling in human lens epithelial cells exposed to H2O2. Int J Mol Med. 2019;44:1515–1522. 31364739 10.3892/ijmm.2019.4283

[32] Wang Q Ju X Chen Y Dong X Luo S Liu H. Effects of L-carnitine against H2O2-induced oxidative stress in grass carp ovary cells (Ctenopharyngodon idellus). Fish Physiol Biochem. 2016;42:845–857. 26701137 10.1007/s10695-015-0179-x

[33] Shafiei G Almasi M Nikzad H Miyan J Mahabadi JA Moshkdanian G. L-carnitine reduces the adverse effects of ROS and up-regulates the expression of implantation related genes in in vitro developed mouse embryos. Theriogenology. 2020;145:59–66. 31986302 10.1016/j.theriogenology.2020.01.008

[34] Haorah J Floreani NA Knipe B Persidsky Y. Stabilization of superoxide dismutase by acetyl-l-carnitine in human brain endothelium during alcohol exposure: Novel protective approach. Free Radic Biol Med. 2011;51:1601–1609. 21782933 10.1016/j.freeradbiomed.2011.06.020 PMC3384514

[35] Chang B Nishikawa M Nishiguchi S Inoue M. L-carnitine inhibits hepatocarcinogenesis via protection of mitochondria. Int J Cancer. 2005;113:719–729. 15499623 10.1002/ijc.20636

[36] Febbraio MA Reibe S Shalapour S Ooi GJ Watt MJ Karin M. Preclinical models for studying NASH-driven HCC: How useful are they? Cell Metab. 2019;29:18–26. 30449681 10.1016/j.cmet.2018.10.012 PMC6326872

[37] Kietzmann T. Metabolic zonation of the liver: The oxygen gradient revisited. Redox Biol. 2017;11:622–630. 28126520 10.1016/j.redox.2017.01.012 PMC5257182

[38] Topcu A Yildiz A Ozkan OF. Effect of L-carnitine on regeneration in experimental partial hepatectomy model in rats. Ulus Travma Acil Cerrahi Derg. 2022;29:9–16. 36588511 10.14744/tjtes.2022.80460 PMC10198347

[39] Kurosaki S Nakagawa H Hayata Y Kawamura S Matsushita Y Yamada T. Cell fate analysis of zone 3 hepatocytes in liver injury and tumorigenesis. JHEP Rep. 2021;3:100315. 34345813 10.1016/j.jhepr.2021.100315 PMC8319533

[40] Matter MS Marquardt JU Andersen JB Quintavalle C Korokhov N Stauffer JK. Oncogenic driver genes and the inflammatory microenvironment dictate liver tumor phenotype. Hepatology. 2016;63:1888–1899. 26844528 10.1002/hep.28487 PMC4874846

[41] Keeton AB Bortoff KD Bennett WL Franklin JL Venable DY Messina JL. Insulin-regulated expression of Egr-1 and Krox20: Dependence on ERK1/2 and interaction with p38 and PI3-kinase pathways. Endocrinology. 2003;144:5402–5410. 12970165 10.1210/en.2003-0592

[42] Zhang J Zhang Y Sun T Guo F Huang S Chandalia M. Dietary obesity-induced Egr-1 in adipocytes facilitates energy storage via suppression of FOXC2. Sci Rep. 2013;3:1476. 23502673 10.1038/srep01476 PMC3600596

[43] Magee N Zhang Y. Role of early growth response 1 in liver metabolism and liver cancer. Hepatoma Res. 2017;3:268–277. 29607419 10.20517/2394-5079.2017.36 PMC5877465

[44] Ueda T Honda M Horimoto K Aburatani S Saito S Yamashita T. Gene expression profiling of hepatitis B- and hepatitis C-related hepatocellular carcinoma using graphical Gaussian modeling. Genomics. 2013;101:238–248. 23485556 10.1016/j.ygeno.2013.02.007

[45] Yoo YD Chiou CJ Choi KS Yi Y Michelson S Kim S. The IE2 regulatory protein of human cytomegalovirus induces expression of the human transforming growth factor beta1 gene through an Egr-1 binding site. J Virol. 1996;70:7062–7070. 8794351 10.1128/jvi.70.10.7062-7070.1996 PMC190757

[46] Hansen HH Pors S Andersen MW Vyberg M Nohr-Meldgaard J Nielsen MH. Semaglutide reduces tumor burden in the GAN diet-induced obese and biopsy-confirmed mouse model of NASH-HCC with advanced fibrosis. Sci Rep. 2023;13:23056. 38155202 10.1038/s41598-023-50328-5 PMC10754821

