# Supplementary material for: Potential utility of l-carnitine for preventing liver tumors derived from metabolic dysfunction–associated steatohepatitis
Source: Hepatol Commun. 2024 Apr 12;8(5):e0425. doi: 10.1097/HC9.0000000000000425 (PMC11019826; doi:10.1097/HC9.0000000000000425)
Supplement: Supplementary file 1 [file hc9-8-e0425-s001.docx]

**Supporting Information for**

**Potential utility of l-carnitine for preventing liver tumors derived from metabolic dysfunction-associated steatohepatitis**

Junyan Lyu^1^, Hikari Okada^2^, Hajime Sunagozaka^2^, Kazunori Kawaguchi^2^, Tetsuro Shimakami^2^, Kouki Nio^2^, Kazuhisa Murai^1^, Takayoshi Shirasaki^1^, Mika Yoshida^1^, Kuniaki Arai^2^, Tatsuya Yamashita^2^, Takuji Tanaka^3^, Kenichi Harada^4^, Toshinari Takamura^5^, Shuichi Kaneko^2^, Taro Yamashita^2^, and Masao Honda^1,2^

Junyan Lyu and Hikari Okada contributed equally to this work.

**Affiliation**

^1^ Department of Clinical Laboratory Medicine, Kanazawa University Graduate School of Medical Sciences, Kanazawa, Japan

^2^ Department of Gastroenterology, Kanazawa University Graduate School of Medical Sciences, Kanazawa, Japan

^3^ Research Center of Diagnostic Pathology, Gifu Municipal Hospital, Gifu, Japan

^4^ Department of Human Pathology, Kanazawa University Graduate School of Medical Sciences, Kanazawa, Japan

^5^ Department of Endocrinology and Metabolism, Kanazawa University Graduate School of Medical Sciences, Kanazawa, Japan

**Supplemental Table 1**

Characteristics of 11 MASH patients and liver histology

­
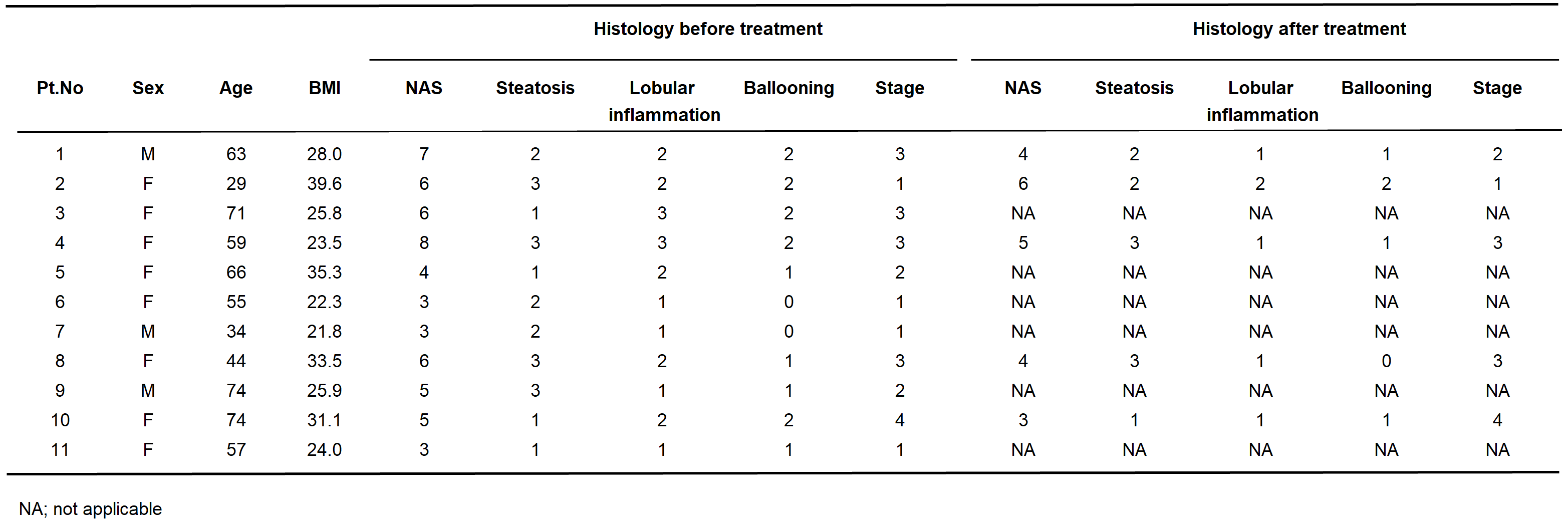


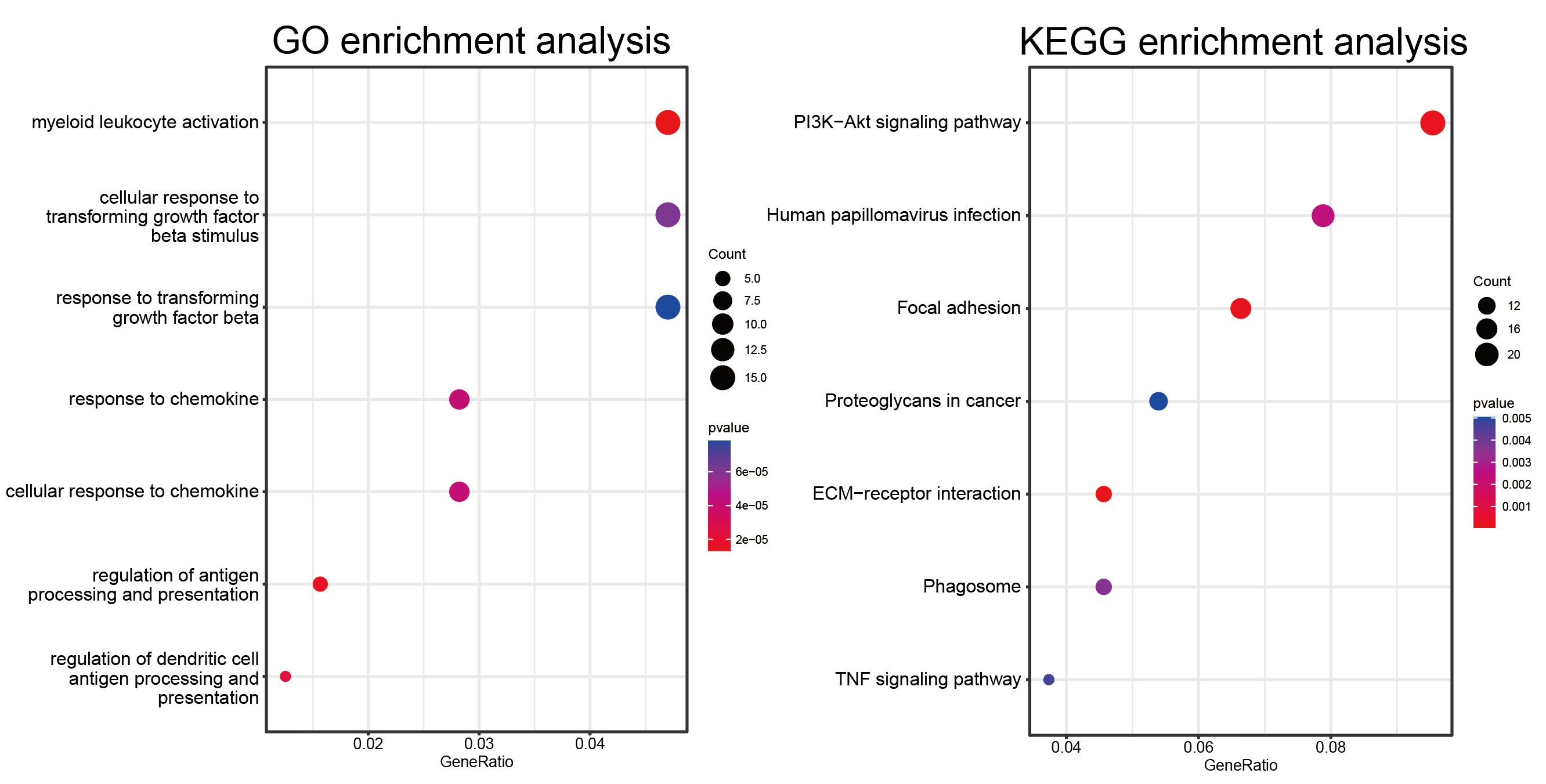


**Supplemental Figure 1.** Gene Ontology (GO)/Kyoto Encyclopedia of Genes and Genomes (KEGG)-based functional analysis of GeneChip data (before vs. after l-carnitine administration).


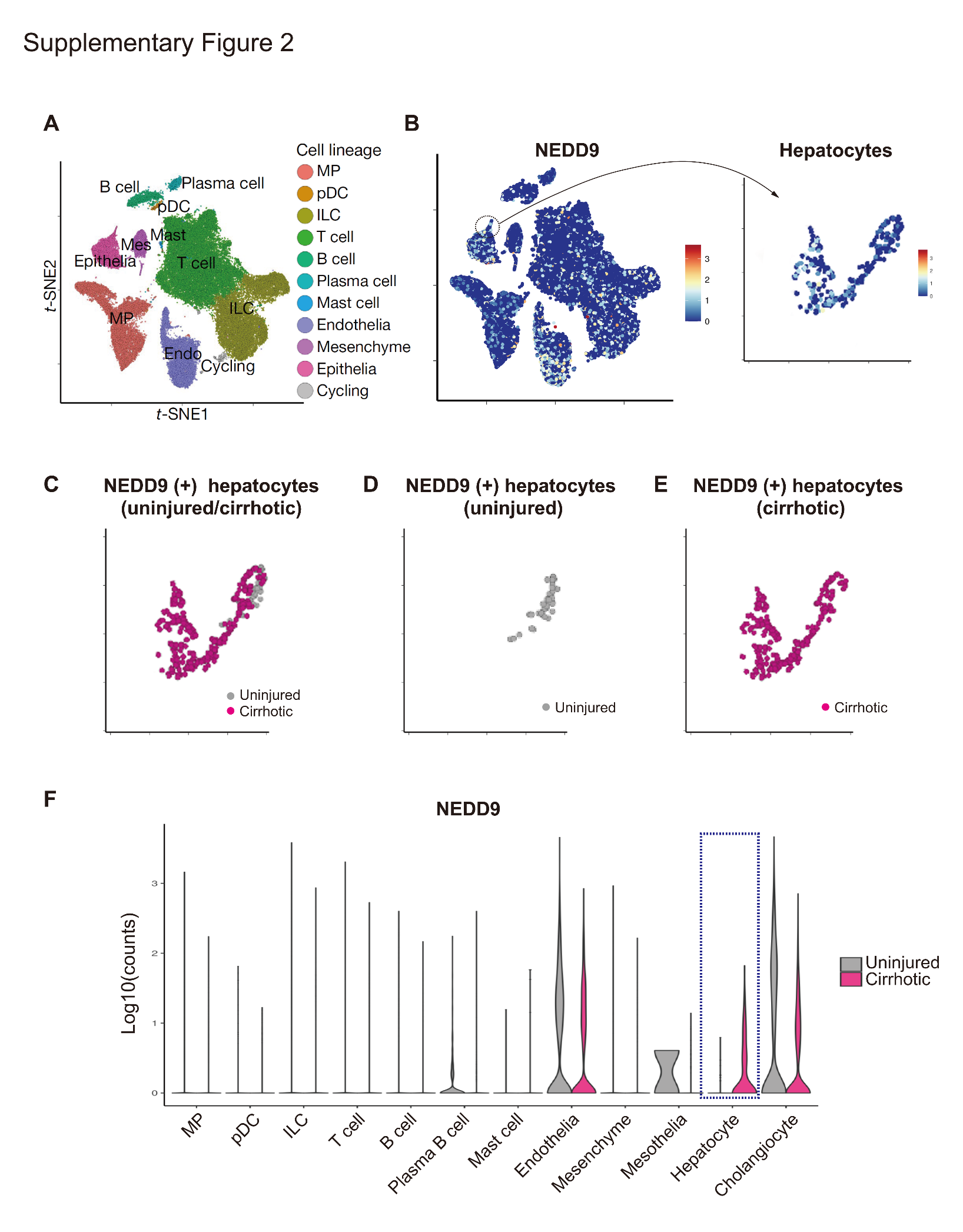


**Supplemental Figure 2. Single-cell analysis of *NEDD9* expression in normal and cirrhotic liver**

A) T-distributed stochastic neighbor embedding (t-SNE) clustering of all lineages obtained from normal and cirrhotic liver. B) *NEDD9* was expressed in the endothelial and epithelial cell clusters (left). Among the clusters of epithelial cells (cholangiocytes and hepatocytes), there are also clusters of hepatocytes (right). C) Combined distributions of *NEDD9*-positive hepatocytes in uninjured and cirrhotic liver. D) Distributions of *NEDD9*-positive hepatocytes in uninjured liver. E) Distributions of *NEDD9*-positive hepatocytes in cirrhotic liver. F) Expression distribution (violin plots) showing normalized *NEDD9* mRNA expression in liver cell clusters in normal and cirrhotic liver. Cluster identities: macrophages (MP), plasmacytoid dendritic cells (pDC), innate lymphoid cells (ILC), T cells, B cells, plasma B cells, mast cells, endothelial cells, mesenchymal cells, mesothelial cells, hepatocytes, and cholangiocytes.

**References**

1. Ramachandran P, Dobie R, Wilson-Kanamori JR, Dora EF, Henderson BEP, Luu NT, Portman JR, et al. Resolving the fibrotic niche of human liver cirrhosis at single-cell level. Nature 2019;575:512-518.

**­­­­**


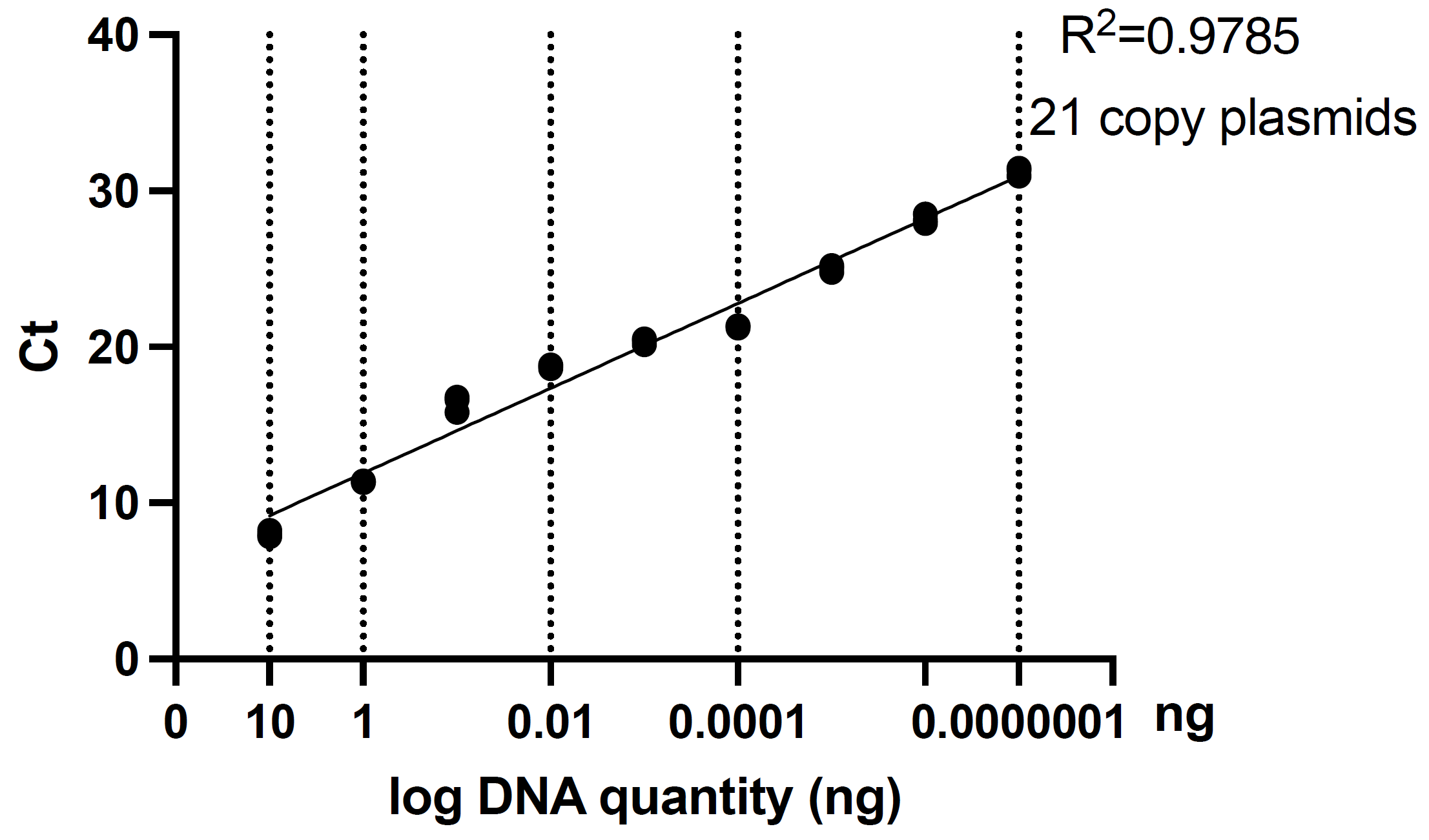


**Supplemental Figure 3. Quantitative measurement of ChIP-precipitated DNA**

The DNA precipitated by anti-EGR1 or control IgG was subsequently analyzed using both PCR and the QuantStudio 3 real-time PCR system (Thermo Fisher Scientific). The primer for the NEDD9 promoter fragment was designed using Primer Premier 5.0 software: (Forward) 5′-TCTCCTTTTCCTGTTCTTTCT-3′, (Reverse) 5′-GTCCTGAACCTTACTCTGTCC-3′, 73 bp. Quantitative analysis was performed using GeneAce SYBR qPCR Mix kit (Nippon Gene, Japan). To quantify the copy number of target sequences and assess primer efficiency, qPCR was performed using serial 10-fold dilutions of a pGL4.10-NEDD9 promoter plasmid with a known molecular weight as a reference to establish a standard curve.
