# Supplementary material for: Potential utility of l-carnitine for preventing liver tumors derived from metabolic dysfunction–associated steatohepatitis
Source: Hepatol Commun. 2024 Apr 12;8(5):e0425. doi: 10.1097/HC9.0000000000000425 (PMC11019826; doi:10.1097/HC9.0000000000000425)
Supplement: Supplementary file 2 [file hc9-8-e0425-s002.docx]

**SUPPLEMENTAL METHODS**

**Cell culture**

The human hepatocellular carcinoma cell line HepG2 was seeded onto collagen-coated plates and maintained in Dulbecco’s Modified Eagle’s Medium (Thermo Fisher Scientific, Waltham, MA) supplemented with 10% fetal bovine serum, 100 U/mL penicillin, and 100 mg/mL streptomycin in a humidified atmosphere at 37°C and 5% CO_2_. Powdered l-carnitine was dissolved in distilled water and then applied to the culture medium at 40, 80, or 160 μmol/L. The medium was replaced with low amino acid medium containing 3 or 5 ng/ mL recombinant human TGF-β1 (R&D Systems, Minneapolis, MN) with or without l-carnitine. After incubation for 24 h, the cells were harvested for analysis. Mouse primary hepatocytes were isolated from 8-week-old male C57BL/6J mice as described previously. (1) All experiments were replicated at least twice. Freshly isolated mouse primary hepatocytes suspended in culture medium were seeded in collagen-coated 6-well plates (IWAKI, Tokyo, Japan), and the culture medium was replaced with medium containing 25 ng/mL recombinant mouse TGF-β1 (R&D Systems) with or without l-carnitine. The cells were harvested for analysis after incubation for 24 h.

**Luciferase reporter assay**

To generate reporter constructs for the luciferase assay, -2,000 to 0 bp DNA fragments of the promoter region of *NEDD9* were inserted into the pGL4.10 vector (Promega Corporation, Madison, WI) using the *Xho*I and *Hind*III sites. Point mutations in the seed region of the predicted EGR1-binding sites were generated using a PrimeSTAR Mutagenesis Basal Kit (Takara Bio, Inc., Shiga, Japan). HepG2 cells were transfected with 500 ng of each reporter construct and 10 ng *Renilla* luciferase control plasmid (pRL-CMV; Promega) with or without 500 ng EGR1 plasmid or empty control plasmid using Lipofectamine 2000 (Thermo Fisher Scientific). After incubation for 24 h, the cells were treated with 10% fetal bovine serum medium. In the dual-luciferase assays, the cells were cultured for 24 h, and luciferase reporter gene expression was measured in cell lysates using the dual luciferase reporter assay system (Promega).

**Overexpression and transfection**

The expression vector for NEDD9 and EGR1 was purchased from GenScript Japan, Inc. (Tokyo, Japan). HepG2 cells were seeded in each well of a 6-well plate. After 24 h, 1 μg plasmid DNA with 2 μL Lipofectamine 2000 (Thermo Fisher Scientific) were added to each well. After 48 h, the cells were harvested for analysis.

**Recombinant proteins and chemicals**

Recombinant human TGF-β1 and mouse TGF-β1 were purchased from R&D Systems (Minneapolis MN). L-carnitine was purchased from Selleck Chemicals (Houston, DE).

**Immunofluorescence staining**

The cells were fixed with methanol and then permeabilized with 0.1% Triton-X 100 in phosphate-buffered saline. A primary anti-EGR1 antibody (1:100 dilution; Cell Signaling Technology) was used at a final concentration of 2 μg/mL in phosphate-buffered saline containing 2% fetal bovine serum at 4°C for 16 h. Incubation with an Alexa Fluor 488-conjugated secondary antibody (Thermo Fisher Scientific) at a 500-fold dilution in phosphate-buffered saline containing 2% fetal bovine serum antibody was performed for 4 h. The chambered cell culture slides were mounted using DAPI, and the cells were viewed using an image analysis system (BIOREVO BZ-9000; KEYENCE).

**Chromatin immunoprecipitation (ChIP) assay**

A ChIP assay was performed using the ChIP IT Express Enzymatic Kit (Active Motif, Carlsbad, CA), following the manufacturer's instructions. Briefly, HepG2 cells were treated with 5 ng/mL rhTGFβ, with or without 160 μM l-carnitine for 24 h, after which the cells were fixed and homogenized. The cell lysates were incubated overnight at 4°C with protein G magnetic beads and either a ChIP-grade anti-EGR1 (Cell Signaling) or control IgG. The DNA precipitated by anti-EGR1 or control IgG was subsequently analyzed using both PCR and the QuantStudio 3 real-time PCR system (Thermo Fisher Scientific). The primer for the NEDD9 promoter fragment was designed using Primer Premier 5.0 software: (Forward) 5′-TCTCCTTTTCCTGTTCTTTCT-3′, (Reverse) 5′-GTCCTGAACCTTACTCTGTCC-3′, 73 bp. The PCR products underwent resolution via electrophoresis on a 3% agarose gel and were visualized using ethidium bromide staining. Quantitative analysis was performed using GeneAce SYBR qPCR Mix kit (Nippon Gene, Japan). To quantify the copy number of target sequences and assess primer efficiency, qPCR was performed using serial 10-fold dilutions of a pGL4.10-NEDD9 promoter plasmid with a known molecular weight as a reference to establish a standard curve.

**Reference**

1. Okada H, Takabatake R, Honda M, Takegoshi K, Yamashita T, Nakamura M, Shirasaki T, et al. Peretinoin, an acyclic retinoid, suppresses steatohepatitis and tumorigenesis by activating autophagy in mice fed an atherogenic high-fat diet. Oncotarget 2017;8:39978-39993.
